# Quantification of Tumor Vessels in Glioblastoma Patients Using Time-of-Flight Angiography at 7 Tesla: A Feasibility Study

**DOI:** 10.1371/journal.pone.0110727

**Published:** 2014-11-21

**Authors:** Alexander Radbruch, Oliver Eidel, Benedikt Wiestler, Daniel Paech, Sina Burth, Philipp Kickingereder, Martha Nowosielski, Philipp Bäumer, Wolfgang Wick, Heinz-Peter Schlemmer, Martin Bendszus, Mark Ladd, Armin Michael Nagel, Sabine Heiland

**Affiliations:** 1 Dept. of Neuroradiology, University of Heidelberg Medical Center, Heidelberg, Baden-Württemberg, Germany; 2 Department of Radiology, German Cancer Research Center (DKFZ), Heidelberg, Baden-Württemberg, Germany; 3 Department of Neurology, University of Heidelberg Medical Center, Heidelberg, Baden-Württemberg, Germany; 4 Department of Neurology, University of Innsbruck, Innsbruck, Austria; 5 Dept. of Medical Physics, German Cancer Research Center (DKFZ), German Cancer Research Center (DKFZ), Heidelberg, Baden-Württemberg, Germany; University of Münster, Germany

## Abstract

***Purpose*:**

To analyze if tumor vessels can be visualized, segmented and quantified in glioblastoma patients with time of flight (ToF) angiography at 7 Tesla and multiscale vessel enhancement filtering.

***Materials and Methods*:**

Twelve patients with newly diagnosed glioblastoma were examined with ToF angiography (TR = 15 ms, TE = 4.8 ms, flip angle = 15°, FOV = 160×210 mm^2^, voxel size: 0.31×0.31×0.40 mm^3^) on a whole-body 7 T MR system. A volume of interest (VOI) was placed within the border of the contrast enhancing part on T1-weighted images of the glioblastoma and a reference VOI was placed in the non-affected contralateral white matter. Automated segmentation and quantification of vessels within the two VOIs was achieved using multiscale vessel enhancement filtering in ImageJ.

***Results*:**

Tumor vessels were clearly visible in all patients. When comparing tumor and the reference VOI, total vessel surface (45.3±13.9 mm^2^ vs. 29.0±21.0 mm^2^ (p<0.035)) and number of branches (3.5±1.8 vs. 1.0±0.6 (p<0.001) per cubic centimeter were significantly higher, while mean vessel branch length was significantly lower (3.8±1.5 mm vs 7.2±2.8 mm (p<0.001)) in the tumor.

***Discussion*:**

ToF angiography at 7-Tesla MRI enables characterization and quantification of the internal vascular morphology of glioblastoma and may be used for the evaluation of therapy response within future studies.

## Introduction

Glioblastoma (glioma of the WHO grade IV) is the most frequent intrinsic brain tumor with a median overall survival from 12 to 14 months [1], [2]. In clinical practice, the suspected diagnosis of glioblastoma is commonly based on contrast-enhanced T1-weighted images that typically reveal a heterogeneously enhanced mass with central necrosis. However, T1-weighted images only visualize the non-specific contrast agent extravasation that is caused by a break-down of the blood brain barrier and do not reflect the vascularization of the glioblastoma [3].

Generally, high grade glioma are characterized by vascular proliferation of tumor vessels that potentially differentiate them from other brain tumors such as low grade glioma [1]. Hence, direct visualization of tumor vessels could potentially contribute to the differential diagnosis of glioblastoma. Beyond this, a quantification of the tumor vasculature could be used for the assessment of antiangiogenic therapies, since an initial normalization of tumor vasculature during therapy response has been reported [4], [5].

Time of flight (ToF) angiography is the most commonly used non-contrast enhanced MR-angiography [6]. However, ToF angiography has not played an important role in the imaging of brain tumors in recent years due to the limited spatial resolution at 1.5 and 3 Tesla [7]. To the best of our knowledge, no MR protocols for ToF angiography at 1.5 or 3 T have been reported that provide a quality sufficient for clinical assessment and vessel segmentation of tumor vessels in brain tumors. Tumor vessels within glioblastoma are supposed to be tortuous, disorganized and highly permeable [8] impeding their detection on ToF angiography at lower field strengths.

Ultra-high-field (UHF) MRI (B0 ≥7 Tesla) has already demonstrated intriguing capabilities and benefits for neuroradiological research [9]. In particular, ToF angiography has been shown to substantially benefit from the increased magnetic field strength of 7 Tesla due to increased signal to noise ratio and longer T1 relaxation times [7], [10]–[13]. Hence, tumor vessels with a diameter below the limit of detection at 3 Tesla may potentially be visualized with 7 Tesla MRI.

In the current study, we assessed the potential of 7 Tesla MRI to visualize tumor vessels in a cohort of glioblastoma patients. Furthermore, we assessed if a postprocessing algorithm based on multiscale vessel enhancement filtering and segmentation in Fiji ImageJ [14] can identify differences between tumor vasculature and non-affected contralateral vessels in terms of total vessel volume, total vessel length, total vessel surface, mean vessel diameter, mean branch length and number of branches.

## Material and Methods

### Patients

**Figure 1:**
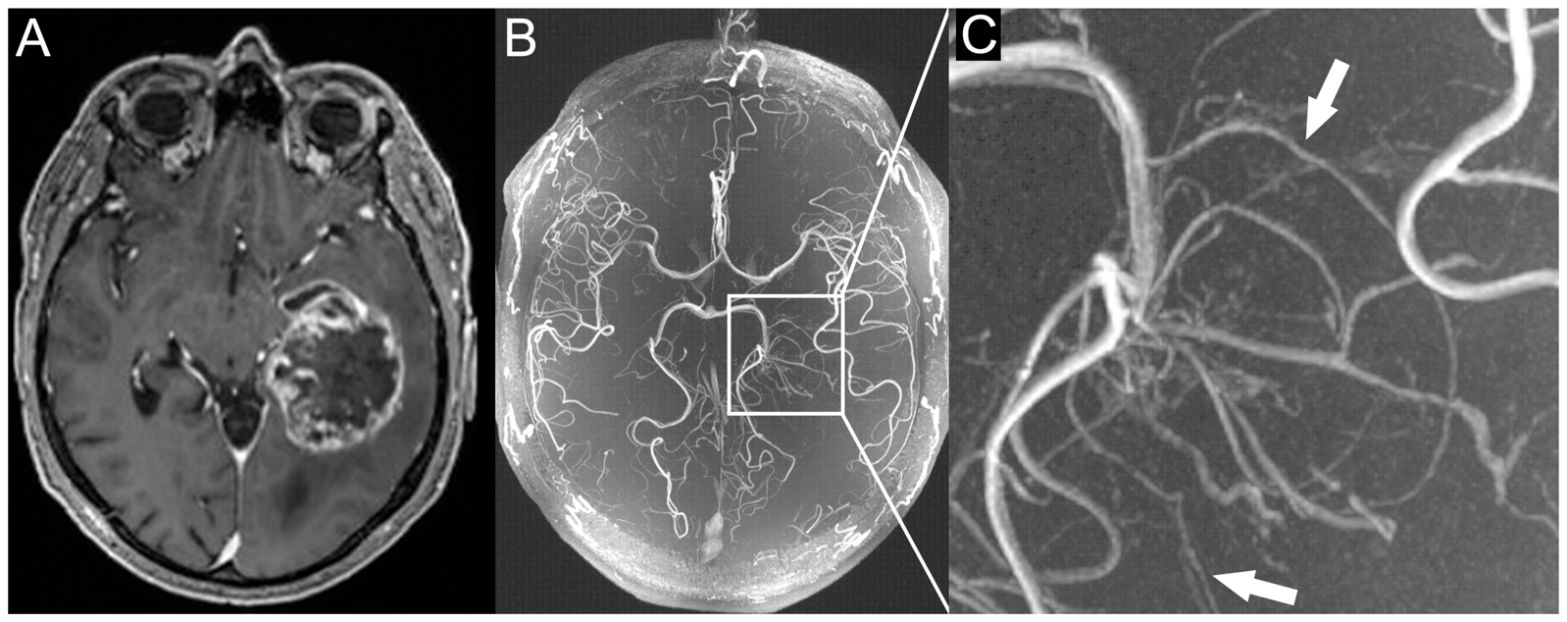
Patient with a left temporal glioblastoma. A) contrast enhanced T1-weighted images at 3 Tesla. B) 7 Tesla ToF angiography maximum intensity projection; enlarged in C). Tumor vessels with multiple branches can clearly be identified within the glioblastoma (white arrows in C). Signal intensity in the tumor vessels is reduced compared to the regular vessels due to signal loss in the irregular and leaky tumor vessels.

This study was approved by the local ethics committee of the University of Heidelberg (MV-341/2008). Before MR examinations, informed written consent was obtained from each patient. Twelve patients with newly diagnosed glioblastoma were prospectively enrolled from the Department of Neurooncology of the University of Heidelberg. Prior to enrollment, all patients were examined with contrast-enhanced 3 Tesla MRI (figure 1A) and informed of the scientific nature of 7 Tesla MR examinations. The patients included 7 men and 5 women ranging in age from 45 to 81 years (median age: 65.5 years). All patients underwent biopsy or resection and the tissue samples were subsequently analyzed at the department of Neuropathology at the University of Heidelberg. All patients were examined prior to biopsy or resection.

### MR Imaging Techniques

A whole-body 7 T MR system (Siemens, Erlangen, Germany) with a 24-channel head coil (Nova Medical, Wilmington, VA) was used for the MR examination. ToF angiography data were acquired using an overlapping multi-slab FLASH sequence with the following imaging parameters: TR = 15 ms, TE = 4.8 ms, flip angle  = 15°, FOV  = 160×210 mm^2^, voxel size: (0.31×0.31×0.40 mm^3^), 3 slabs, total acquisition time: 6 min 34 s.

Additionally, contrast enhanced T1-weighted scans (TE = 4 ms, TR = 1710 ms, flip angle  = 15°, acquisition matrix  = 512×512×164, and voxel size  = 0.5 mm×0.5 mm×1.3 mm) that were acquired within clinical routine on a whole-body 3 T MR system (Trio or Verio, Siemens, Erlangen, Germany) were used for morphological tumor assessment.

### Image Analysis Tools

#### Fiji ImageJ

Fiji is an image-processing package and can be described as a distribution of ImageJ (and ImageJ2) together with Java, Java3D and many plugins. The main focus of Fiji is to assist research in life sciences [15].

#### Multiscale Vessel Enhancement Filtering by Frangi et al

Automatic segmentation based on the Multiscale Vessel Enhancement Filtering method developed by Frangi et al. [14] has been successfully applied to fundus images of the eye [16] and brain angiography images at 1.5 Tesla [17].

The filter obtains vesselness measures for each voxel (i.e. highlights tubelike structures) by analyzing eigenvalues of the Hessian matrix (*H*), which describes the curvature as a function of direction. *H* was calculated for each pixel and consisted of the second-order derivatives of the corresponding input image I(x,y,z). Due to the input image being three-dimensional, *H* was defined as
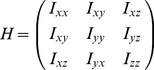
(1)


The Hessian matrix H_0,s_ is calculated at each pixel position *x_0_* and scale *s*. In Frangi's method, *s* uses the standard deviation (*σ*) of Gaussians to approximate the second-order derivatives. At each pixel position *x_0_*, a vesselness feature V_0_(s) is calculated from the eigenvalues *λ1*<*λ2* of the Hessian matrix H_0,s_ using a “dissimilarity measure” *R_B_* and “second order structuredness” *S*.
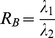


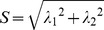


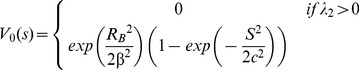
(2)


Filter sensitivity is controlled through the constants β and *c*. *R_B_* accounts for the deviation from blob-like structures and S helps to differentiate between noise and background, since background pixels have a small magnitude of derivatives and, therefore, small eigenvalues [16].

#### 3D Objects Counter

The 3D Objects Counter is a Fiji ImageJ Plugin written in Java and was first applied in light microscopy [18], [19]. Applications in medical research, until now, were mostly limited to microscopy [20]–[22]. To the best of our knowledge, it has not been utilized for blood vessel segmentation yet. The user is first asked to set a threshold on the image. Pixels with intensities above the threshold will be considered as the object's pixels (in our case, vessels) while other pixels will be considered as being part of the background. Then, the plugin detects objects by performing connectivity analysis and therefore finding pixels which are connected to each other. Finally, statistics for each object are computed, including the number of the object's voxels and the number of surface voxels. These can further be used to calculate the object's volume and surface by multiplication with the MRI sequence-specific voxel size.

#### Skeletonization and Skeleton Analysis

Skeletonization was necessary for the calculation of the number of branches per vessel und their respective length and has already been successfully applied for blood vessels [23]. Therefore, the image was skeletonized with the Fiji ImageJ Plugin “Skeletonize3D” [24] which is based on a 3D thinning algorithm by Lee et al. [25] The Algorithm finds centerlines in structures (in our case, vessels) and erodes the surface iteratively until only the skeleton as a binary image remains. Subsequently, the skeletonized images were analyzed with the plugin “AnalyzeSkeleton” [15], [26] and numbers of branches per vessel and branch length were computed.

#### Automatic Segmentation Algorithm

Vessel segmentation and quantification were performed automatically with an in-house developed macro for Fiji ImageJ.

**Figure 2:**
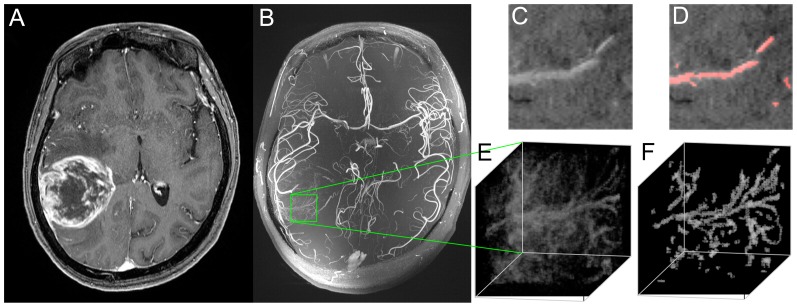
Demonstration of multiscale vessel enhancement filtering. A) Contrast enhanced T1 weighted images at 3 Tesla of a patient with right temporal glioblastoma. B) 7 Tesla ToF angiography maximum intensity projection. Within the selected VOI (E) (representative slide in (C)), vessels are automatically detected and visualized (F) (representative slide with red colored vessels in (D)).

First, vessels were enhanced by applying the multiscale vessel enhancement filter described by Frangi et al. [14] to the TOF image series. Then, images were thresholded with an individual threshold and objects with a minimum size of 4 voxels were counted utilizing the Fiji ImageJ 3D Objects Counter [18], [19]. The threshold was manually set to a value at which vessels were detected while background signal and other vessel-like structures and artefacts were ignored. Fourteen volumes of interest (VOIs) were processed using the same threshold (2200 arb. unit) while ten VOIs needed a higher threshold (3975±2942 arb. unit) due to a higher number of artifacts. Therefore, the correctness of vessel detection in every patient was validated by a Neuroradiologist (AR) using a 2D composite image (Fig. 2, C and D) and a 3D projection (Fig. 2, E and F). Finally, the thresholded image was skeletonized for analysis of the number of branches and their respective length [15], [24]–[26].

A VOI was placed within the border of the contrast enhancing part on T1-weighted images of the glioblastoma and a reference VOI was placed in the non-affected contralateral white matter. Tumor VOI sizes varied due to different sizes of tumor regions and were, on average, smaller than the control regions (9.8±4.1 cm^3^ vs. 13.0±5.2 cm^3^). Values of total vessel length, vessel surface, vessel volume and number of branches were standardized to a volume of one cubic centimeter for comparability. The postprocessing algorithm is visualized in figure 2. To assess the variability of values within the tumor and the contralateral white matter, three VOIs were placed exemplarily in the tumor of one patient and in the contralateral white matter (occipital, parietal and frontal).

### Statistical Analysis

Statistical analysis was conducted with R, version 3.0.2 and plots were visualized with ggplot2 [27], [28]. All acquired parameters within the tumor and the contralateral hemisphere were compared using the Wilcoxon signed-rank test. P values <0.05 were deemed statistically significant.

## Results

Tumor vessels could be visualized clearly on the ToF angiography of all included 12 patients (e.g. figure 2B).

**Figure 3:**
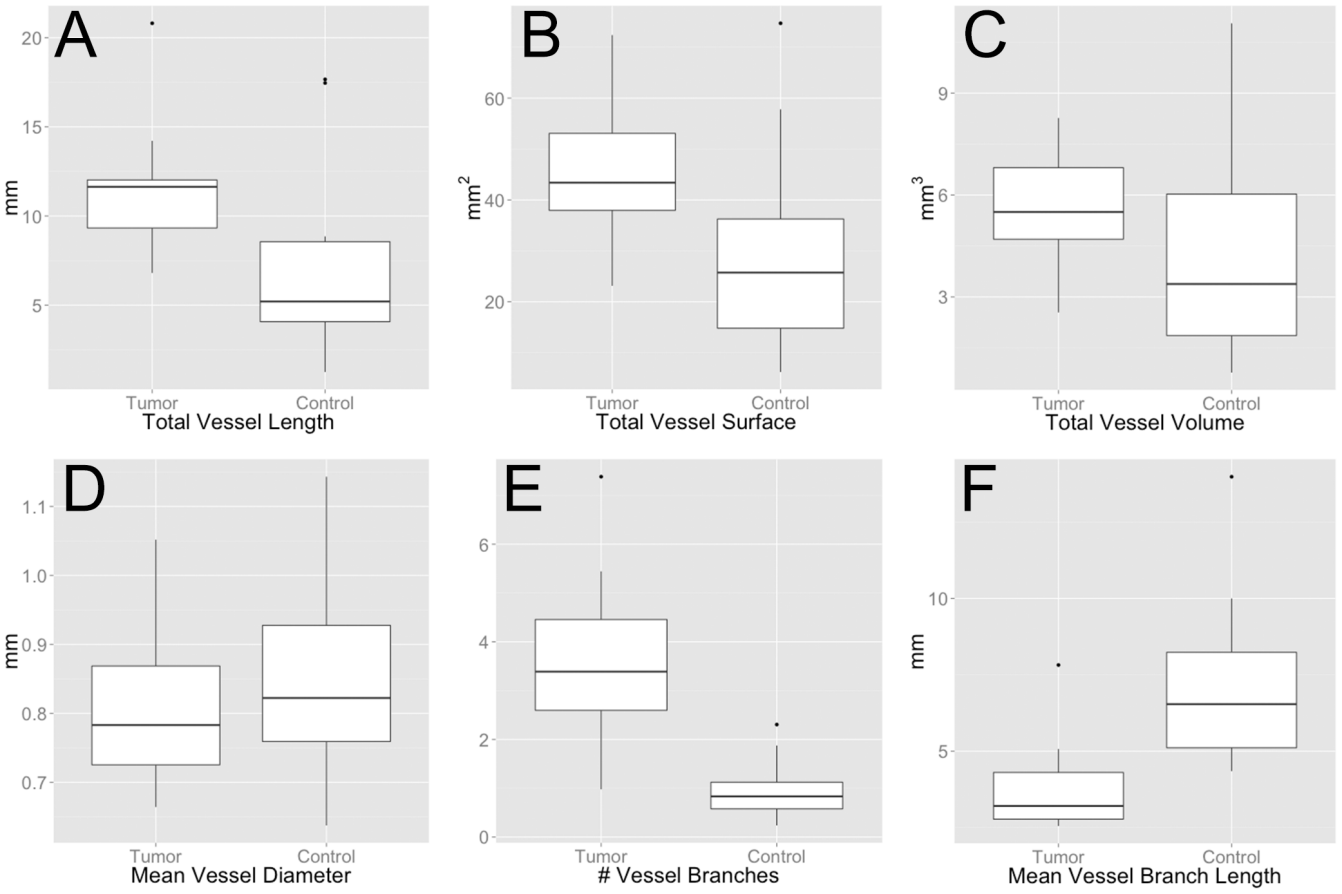
Box plots of assessed parameters within tumor VOI and control VOI in the contralateral white matter. Total vessel length (A) (p<0.064), total vessel surface (B) (p<0.035), vessel volume (C) (p<0.078) and number of vessel branches (E) (p<0.001) per cubic centimeter are increased within the tumor compared to contralateral white matter. In contrast mean vessel diameters (D) (p<0.064) and average branch length of vessels (F) (p<0.001) are decreased in the tumor.

**Figure 4:**
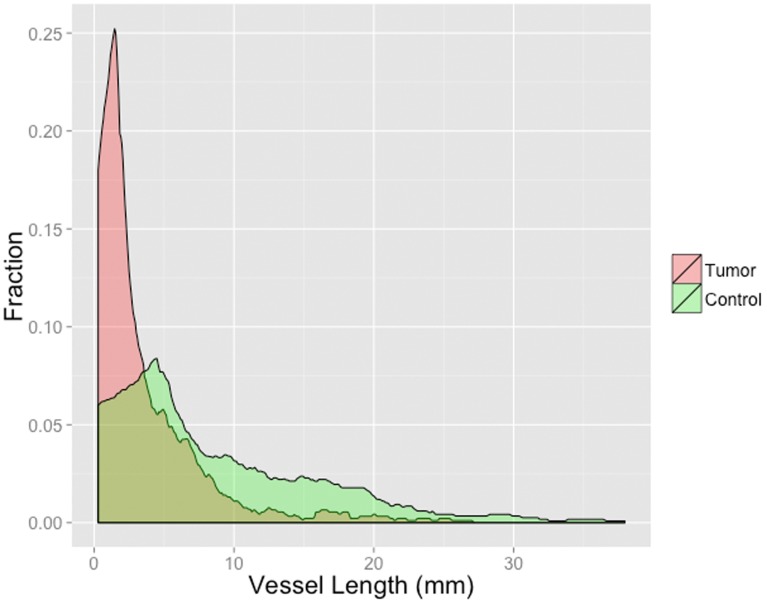
Histogram of mean vessel branch length; the area under the curve is normalized to 1.

When comparing tumor and reference VOI, total vessel surface (45.3±13.9 mm^2^ vs. 29.0±21.0 mm^2^ (p<0.035)) and number of branches (3.5±1.8 vs. 1.0±0.6 (p<0.001) per cubic centimeter were significantly higher in the tumor, while mean vessel branch length was significantly lower (3.8±1.5 mm vs 7.2±2.8 mm (p<0.001)) in the tumor. Total vessel length per cubic centimeter (11.363±3.752 mm vs. 7.175±5,429 mm (p<0.064) and total vessel volume per cubic centimeter (5.633±1.596 mm^3^ vs. 4.1073±3.103 mm^3^ (p<0.078)) tended to be higher while mean vessel diameter (0.803±0.117 mm vs 0.8541±0.146 mm (p<0.064)) tended to be lower in the tumor, without reaching statistical significance. All results are summarized in figure 3. Results for mean vessel branch length are visualized additionally in a histogram (figure 4).

The three different VOIs within the tumor and the contralateral white matter that were exemplarily calculated in one patient to assess the variability of values yielded 12.2±1.8 mm (tumor) and 5.9±0.5 mm (contralateral white matter) for total vessel length; 43.9±4.9 mm^2^ (tumor) and 24.5±4.5 mm^2^ (contralateral white matter) for total vessel surface; 4.8±0.7 mm^3^ (tumor) and 2.5±0.4 mm^3^ (contralateral white matter) for total vessel volume; 0.7±0.07 mm (tumor) and 0.7±0.03 mm (contralateral white matter) for vessel diameter; 4.2±0.4 (tumor) and 2.0±0.08 (contralateral white matter) for number of branches; 2.9±0.22 (tumor) and 3.9±0.7 (contralateral white matter) average branch length.

## Discussion

As a principal finding of our study, we proved the feasibility of tumor vessel imaging in glioblastoma with ToF angiography at 7 Tesla. Furthermore, we showed that the identified tumor vessels can be quantified in terms of total vessel volume, total vessel length, total vessel diameter, mean vessel diameter, mean branch length and number of branches using multiscale vessel enhancement filtering and segmentation.

Generally, a slab selective 3 D steady state gradient echo sequence with a pulse repetition time much shorter than the T1 relaxation time of static tissue is used in 3D-ToF angiography, combined with a sufficiently large flip angle [29]. Under this steady state regime, fully relaxed blood flowing into the excitation slab provides a strong MR signal while the background tissue is strongly reduced [29]. Interpretation of ToF angiography is often impaired due to imaging artifacts such as signal misregistration or intravoxel phase dispersion caused by pulsatile, complex or turbulent flow patterns in curving and branching vessel geometry [30].

This especially holds true for glioblastoma vessels that are tortuous, disorganized, highly permeable and characterized by abnormalities in their endothelial wall, pericyte coverage and basement membrane [8].

Furthermore, it was shown in animal models that the leakiness of the tumor vessels, along with other morphological abnormalities of the network, results in a red blood cell velocity that is one to three orders of magnitude lower than in the surrounding normal pial vessels [31]. Due to these pathologic properties, tumor vessels within glioblastoma usually cannot be visualized with ToF angiography at lower field strengths.

ToF angiography directly benefits from the increased field strength at ultra high field MRI such as 7 Tesla [10], [12], [32]. Main contributions for this are an increase in intrinsic signal to noise ratio and longer T1 relaxation constants in brain tissues [11], [29]. Patient studies using ToF angiography at 7 T, focusing mainly on aneurysma and stroke patients [33], [34], have already been conducted, proving the benefit of ToF angiography at 7 T compared to lower field strengths.

To the best of our knowledge, this is the first study that proves the feasibility of tumor vessel imaging in a glioblastoma patient cohort at 7 Tesla. Within all 12 glioblastoma patients, tumor vessels could be clearly visualized. We therefore hypothesize 7 Tesla MRI to be a promising imaging modality for the detection of tumor vessels in glioblastoma. The multiscale vessel enhancement filtering and segmentation algorithm revealed that the total vessel surface and the number of branches of all vessels are significantly increased within the tumor, while mean vessel branch length was significantly decreased. Even though the increase of total vessel length and volume within the tumor did not reach statistical significance, a tendency became apparent. These results are in line with the above mentioned histopathological features of glioblastoma, presenting increased vascularization of tortuous and disorganized vessels. In contrast, the found tendency of glioblastoma vessels to display a smaller mean diameter is contradictory to histopathological studies that reported glioblastoma vessels to have a larger diameter [8]. However, it has to be taken into account that ToF angiography tends to underestimate the diameter of tumor vessels due to a decreased blood flow in the tumor vessels accompanied by an accelerated signal loss. This property of ToF angiography might be one reason for the found tendency of a decreased mean vessel diameter within the tumor.

In clinical routine, tumor vasculature is commonly assessed indirectly by MR perfusion imaging [3]. Currently, vascular blood volume and vascular permeability parameters derived from MR perfusion are considered surrogate imaging markers of angiogenesis [35]. Nevertheless, perfusion imaging of brain tumors provides multiple pitfalls [36] and finally only presents an indirect parameter for tumor vascularization. Although the visualization of tumor vessels is principally feasible with the help of digital subtraction angiography, this technique is supposed to be obsolete within tumor-imaging as it may be associated with multiple side effects and should not be used on a regular basis for follow up examinations. Therefore, the introduced multiscale vessel enhancement filtering algorithm of ToF angiography at 7 Tesla might present an alternative method that enables a direct quantification of the tumor vasculature.

A further limitation of the direct visualization of tumor vessels with ToF angiography is that only vessels with a diameter of approximately 300 µm can be visualized. In contrast, advanced perfusion post-processing techniques using vessel architectural imaging have been reported to estimate microvascular vessel caliber up to capillary size of 3.5 µm [4], [37]. Thus, a comprehensive assessment of the tumor vasculature within future studies could use a combined approach: direct visualization of larger tumor vessels with ToF angiography at 7 Tesla and indirect calculation of microvessel density using advanced perfusion postprocessing techniques.

A limitation of the applied ToF protocol in the current study is that due to specific absorption rate constraints no radiofrequency pulses were used to saturate the venous signal. This might result in a misinterpretation of venous structures as arteries. Specific absorption strategies enabling the use of venous saturation at 7 Tesla might help to overcome this shortcoming within future studies [10].

Another specific problem at 7 Tesla derives from shorter radiofrequency wavelengths that result in significantly higher spatial heterogeneity in transmit B1 magnitude, potentially yielding contrast and signal variations [29]. The acquisition of ToF angiography might be optimized within future studies by the use of advanced B1 shimming techniques that limit this shortcoming, such as recently introduced by Schmitter et al [29].

Finally, the quantification of the tumor vessels with ToF angiography obviously depends on the underlying vessel detection algorithm. The applied algorithm has already been conducted for vessel segmentation in fundus images of the eye [16] and for brain vessel detection in ToF angiography at 1.5 Tesla [17]. Like other vessel enhancement filters, the multiscale vessel enhancement filtering also enhances non-vessel structures, which have similar characteristics to vessels. Enhancement of non-vascular structures is not specific to these filters [38], as designing a mathematical model of the vasculature with 100% specificity is not possible. The filters can also decrease the visualization of vascular structures such as aneurysms that do not match the filter assumptions. The plugin 3D Objects Counter has not been used for blood vessel segmentation yet. Therefore, each segmentation was additionally evaluated by a neuroradiologist (AR) and yielded similar results to the maximum intensity projection. Furthermore, the exact combination of these tools has never been applied before. However, in our experience, the multiscale vessel enhancement filtering algorithm can provide vessel segmentation comparable to the original maximum intensity projection images [17].

The additional assessment of one patient with three different VOIs in the tumor and in the contralateral white matter yielded comparable values and small standard deviations within the tumor and the contralateral white matter. Since the vascularization should be comparable in different parts of the white matter, this result was expected for the white matter and underlines the robustness of the applied algorithm. For the interpretation of the comparable values within the tumor, it is important to point out that the VOI covered a large part of the tumor, impeding the identification of different areas of vascularization within the tumor. Future studies might use more sophisticated segmentation methods that overcome the shortcomings connected to the VOI based analysis.

Ultimately, we acknowledge that the included number of patients is relatively small and that studies with larger patient numbers should be conducted to confirm our findings. These trials are currently hampered by the limited availability of 7 Tesla scanners for centers treating brain tumor patients. However, the number of 7 Tesla sites is constantly increasing and the potential clinical use of the introduced technique might be investigated in collaborative studies.

Such a collaborative study could be the investigation of therapy response assessment in glioblastoma patients treated with anti-angiogenic medication. In a preclinical mouse model using intravital 2D photon microscopy, it has been shown that Bevacizumab induced a decrease in vascular volume and reduced the number of vascular branching points [39]. The differentiation of patients who benefit from anti-angiogenic treatment and patients who only suffer from side effects currently remains an unsolved problem within daily clinical decision making. Hence, imaging biomarkers that stratify responders from non-responders are highly needed. Since anti-angiogenic agents are supposed to normalize the tumor vasculature, [40] the proposed method may contribute to the required differentiation of responders from non-responders and should be further evaluated in future clinical studies.

## Conclusion

Multiscale vessel enhancement filtering of ToF angiography at 7-Tesla MRI enables characterization and quantification of the internal vascular morphology of glioblastoma. It can potentially be used for the evaluation of therapy response in anti-angiogenic therapies within future clinical studies.
